# Transforming growth factor-β signaling pathway-associated genes *SMAD2* and *TGFBR2* are implicated in metabolic syndrome in a Taiwanese population

**DOI:** 10.1038/s41598-017-14025-4

**Published:** 2017-10-19

**Authors:** Eugene Lin, Po-Hsiu Kuo, Yu-Li Liu, Albert C. Yang, Shih-Jen Tsai

**Affiliations:** 1 Graduate Institute of Biomedical Sciences, China Medical University, Taichung, Taiwan; 2 Vita Genomics, Inc., Taipei, Taiwan; 3 TickleFish Systems Corporation, Seattle, WA USA; 4 Department of Public Health, Institute of Epidemiology and Preventive Medicine, National Taiwan University, Taipei, Taiwan; 5 Center for Neuropsychiatric Research, National Health Research Institutes, Miaoli County, Taiwan; 6 Department of Psychiatry, Taipei Veterans General Hospital, Taipei, Taiwan; 7 Division of Psychiatry, National Yang-Ming University, Taipei, Taiwan

## Abstract

The transforming growth factor-β (TGF-β) signaling pathway and its relevant genes have been correlated with an increased risk of developing various hallmarks of metabolic syndrome (MetS). In this study, we assessed whether the TGF-β signaling pathway-associated genes of SMAD family member 2 (*SMAD2*), *SMAD3*, *SMAD4*, transforming growth factor beta 1 (*TGFB1*), *TGFB2*, *TGFB3*, transforming growth factor beta receptor 1 (*TGFBR1*), and *TGFBR2* are associated with MetS and its individual components independently, through complex interactions, or both in a Taiwanese population. A total of 3,000 Taiwanese subjects from the Taiwan Biobank were assessed. Metabolic traits such as waist circumference, triglyceride, high-density lipoprotein cholesterol, systolic and diastolic blood pressure, and fasting glucose were measured. Our results showed a significant association of MetS with the two single nucleotide polymorphisms (SNPs) of *SMAD2* rs11082639 and *TGFBR2* rs3773651. The association of MetS with these SNPs remained significant after performing Bonferroni correction. Moreover, we identified the effect of *SMAD2* rs11082639 on high waist circumference. We also found that an interaction between the *SMAD2* rs11082639 and *TGFBR2* rs3773651 SNPs influenced MetS. Our findings indicated that the TGF-β signaling pathway-associated genes of *SMAD2* and *TGFBR2* may contribute to the risk of MetS independently and through gene–gene interactions.

## Introduction

The transforming growth factor-β (TGF-β) signaling pathway, a major intercellular signaling pathway in animal cells, plays a key role in regulating many aspects of cellular processes such as cell proliferation, cell differentiation, extracellular matrix production, embryonic development, cell adhesion, and cell apoptosis^1,2^. Given its widespread functionality, abnormalities in the TGF-β signaling pathway have been found to lead to diverse human diseases such as hypertension, hyperlipidemia, atherosclerosis, renal and cardiac fibrosis, and cancer, which are in turn associated with metabolic syndrome (MetS)^3–6^. The TGF-β family members include TGF-β, TGF-β type I receptor, TGF-β type II receptor, SMAD family member 2 (SMAD2), SMAD3, SMAD4, and other relevant proteins^1,2^. TGF-β ligands first bind to type II receptors, which then form a ligand–receptor complex with type I receptors^1,2^. The TGF-β type I receptor directly activates intracellular SMAD2 and SMAD3 proteins through their phosphorylation, which then mediate TGF-β signals. SMAD2 and SMAD3 proteins also target a common mediator, the SMAD4 protein, to form a heteromeric SMAD complex, which is subsequently translocated to the cell nucleus. The genes involved in the TGF-β signaling pathway include the *SMAD2*, *SMAD3*, *SMAD4*, transforming growth factor beta 1 (*TGFB1*), *TGFB2*, *TGFB3*, transforming growth factor beta receptor 1 (*TGFBR1*), and *TGFBR2* genes.

The *SMAD2*, *SMAD3*, and *SMAD4* genes are located on chromosome 18q21.1, 15q22.33, and 18q21.2, respectively. The proteins encoded by the *SMAD2*, *SMAD3*, and *SMAD4* genes belong to the SMAD protein family, which mediates TGF-β signals^1,2^. By using imputation based on the 1000 Genomes Project, a genome-wide association study (GWAS) meta-analysis of the data from the CARDIoGRAMplusC4D Consortium indicated that the rs56062135 single nucleotide polymorphism (SNP) in the intron region of the *SMAD3* gene is associated with coronary artery disease (CAD)^7^. A subsequent study by Turner *et al*. indicated that the *SMAD3* rs17293632 SNP, which is in strong linkage disequilibrium (LD) with the *SMAD3* rs56062135 SNP, may contribute to susceptibility to CAD^8^. In several animal studies, the TGF-β/SMAD signaling pathway has been correlated with the hallmarks of MetS, including insulin resistance, obesity, diabetes, and lipid metabolism^9–12^.

The *TGFB1*, *TGFB2*, and *TGFB3* genes are located on chromosome 19q13.2, 1q41, and 14q24.3, respectively. The three structurally similar isoforms (TGF-β1, TGF-β2, and TGF-β3) encoded by the *TGFB1*, *TGFB2*, and *TGFB3* genes are secreted ligands that belong to the TGF-β protein superfamily; these ligands bind to various TGF-β receptors to recruit and activate the SMAD protein family^1,2^. Studies have shown that *TGFB1* SNPs are associated with various diseases such as CAD, stroke, chronic kidney diseases, and inflammatory diseases^13^. Evidence also supports the association of *TGFB2* SNPs with end-stage renal disease^14^ and conotruncal heart defects^15^. Moreover, research has indicated that the *TGFB3* rs11466414 SNP increases the risk of pregnancy-induced hypertension in a Hispanic population^16^.

The *TGFBR1* and *TGFBR2* genes are located on chromosome 9q22.33 and 3p24.1, respectively. The proteins encoded by the *TGFBR1* and *TGFBR2* genes belong to the TGF-β receptor subfamily; these proteins form a heterodimeric complex that binds to TGF-β proteins^1,2^. A previous study demonstrated that the *TGFBR2* rs9838682 SNP is likely to influence the risk of sudden cardiac arrest in the setting of CAD in a Caucasian population^17^. In addition, the *TGFBR2* rs6785358 SNP has been linked to a predisposition to congenital heart defects in a Chinese population^18,19^. Moreover, it has been observed that the overexpression of the TGF-β type I receptor and TGF-β type II receptor may be associated with autophagy and fibrogenesis in human heart diseases^20^. An animal study suggested that the deletion of the *Tgfbr2* gene in hepatocytes influences systemic insulin resistance and body weight gain in mice during the development of nonalcoholic fatty liver disease, which is the hepatic manifestation of MetS^12^. Furthermore, several meta-analyses have suggested that *TGFBR1* SNPs are associated with increased risks of breast, ovarian, and colorectal cancer^21,22^.

Based on the aforementioned findings, it is speculated that the TGF-β signaling pathway and its relevant genes play a key role in the development of MetS. Thus, we hypothesized that TGF-β signaling pathway-associated genes, namely *SMAD2*, *SMAD3*, *SMAD4*, *TGFB1*, *TGFB2*, *TGFB3*, *TGFBR1*, and *TGFBR2* genes, may be linked to MetS. To the best of our knowledge, scant human studies have investigated the influence of these genes on MetS. Therefore, we conducted an association study to determine the relationships between susceptibility to MetS and SNPs in the *SMAD2*, *SMAD3*, *SMAD4*, *TGFB1*, *TGFB2*, *TGFB3*, *TGFBR1*, and *TGFBR2* genes. We also assessed the effects of potential gene–gene interactions on MetS.

## Results

Table 1 describes the demographic and clinical characteristics of the study population. First, we investigated the association between MetS and 8 TGF-β signaling pathway-associated genes, namely *SMAD2*, *SMAD3*, *SMAD4*, *TGFB1*, *TGFB2*, *TGFB3*, *TGFBR1*, and *TGFBR2* genes. Based on LD, we filtered SNPs and selected 141 tag SNPs (Supplementary Table S2). Among the 141 tag SNPs assessed in this study (Supplementary Table S3), there were 20 tag SNPs, among those SNPs present in the *SMAD2*, *SMAD3*, *SMAD4*, *TGFB2*, *TGFB3*, and *TGFBR2* genes, which showed evidence of an association (P < 0.05) with MetS (Table 2).

**Table 1:** Demographic and clinical characteristics of study subjects.

Characteristic	Without MetS	With MetS	P value
No. of subjects (n)	2467	533	
Age (years)	48.3 ± 10.9	53.3 ± 10.1	<0.0001
Sex (female; %)	53.9%	51.8%	0.371
Waist circumference (female; cm)	79.0 ± 8.4	90.5 ± 8.9	<0.0001
Waist circumference (male; cm)	85.6 ± 7.5	97.5 ± 6.8	<0.0001
Triglyceride (mmol/L)	1.13 ± 0.65	2.22 ± 1.61	<0.0001
HDL (mmol/L)	1.45 ± 0.33	1.13 ± 0.24	<0.0001
Systolic blood pressure (mmHg)	112.8 ± 15.6	126.9 ± 17.8	<0.0001
Diastolic blood pressure (mmHg)	70.2 ± 10.3	77.3 ± 11.0	<0.0001
Fasting glucose (mmol/L)	5.21 ± 0.85	6.34 ± 1.96	<0.0001

HDL cholesterol = high-density lipoprotein cholesterol, MetS = metabolic syndrome.

Data are presented as mean ± standard deviation.

**Table 2:** Covariate-adjusted odds ratio analysis of the relationship between MetS and 20 tag SNPs in the TGF-β signaling pathway-associated genes of *SMAD2*, *SMAD3*, *SMAD4*, *TGFB2*, *TGFB3*, and *TGFBR2* with evidence of an association (P < 0.05).

				Additive model			Dominant model			Recessive model		
Gene	SNP	A1	A2	OR	95% CI	P	OR	95% CI	P	OR	95% CI	P
*SMAD2*	rs1792684	A	G	1.17	1.01–1.35	**0.0352**	1.08	0.89–1.31	0.4477	1.36	1.04–1.77	**0.0229**
	rs74430094	C	T	1.73	1.01–2.99	**0.0480**	1.32	0.99–1.76	0.0562	2.92	0.98–8.69	0.0536
	rs11082639	C	T	1.66	1.32–2.08	**1.4 × 10** ^**−5**^	1.05	0.86–1.28	0.6611	2.82	1.80–4.43	**6.6 × 10** ^**−6**^
	rs4940086	C	T	0.79	0.68–0.91	**0.0015**	0.76	0.62–0.92	**0.0052**	0.70	0.54–0.92	**0.0102**
	rs2000709	A	G	1.48	1.15–1.90	**0.0020**	1.01	0.82–1.24	0.9567	2.24	1.37–3.68	**0.0013**
*SMAD3*	rs28417316	G	A	1.32	0.67–2.59	0.4207	1.40	1.08–1.80	**0.0099**	1.66	0.43–6.36	0.4637
	rs7181878	A	G	1.16	1.01–1.34	**0.0421**	1.20	0.98–1.46	0.0725	1.25	0.96–1.62	0.0989
	rs67614233	T	C	1.25	1.00–1.55	**0.0469**	1.12	0.92–1.37	0.2455	1.51	0.99–2.32	0.0568
	rs2289263	G	T	1.17	1.01–1.35	**0.0340**	1.00	0.83–1.22	0.9775	1.43	1.10–1.86	**0.0085**
*SMAD4*	rs17811426	T	C	0.88	0.76–1.02	0.0999	1.00	0.81–1.22	0.9625	0.75	0.57–0.97	**0.0315**
*TGFB2*	rs2799086	T	C	0.94	0.78–1.13	0.5320	0.82	0.67–0.99	**0.0378**	0.98	0.68–1.40	0.9014
*TGFB3*	rs3917211	C	T	1.08	0.93–1.26	0.3021	1.26	1.04–1.53	**0.0197**	1.03	0.77–1.37	0.8500
	rs2284791	C	G	1.11	0.96–1.28	0.1661	1.40	1.13–1.72	**0.0018**	0.97	0.76–1.25	0.8127
	rs3917201	C	T	1.18	1.03–1.36	**0.0190**	1.46	1.16–1.84	**0.0012**	1.06	0.85–1.32	0.6010
	rs4252328	T	C	1.15	1.00–1.32	0.0553	1.30	1.05–1.61	**0.0151**	1.12	0.88–1.41	0.3682
	rs2268626	C	T	1.21	1.04–1.40	**0.0142**	1.20	0.99–1.46	0.0643	1.37	1.03–1.81	**0.0301**
*TGFBR2*	rs1991657	C	T	0.84	0.67–1.05	0.1309	0.80	0.65–0.97	**0.0241**	0.76	0.49–1.19	0.2320
	rs3773651	G	A	1.50	1.04–2.15	**0.0285**	1.59	1.28–1.98	**3.1 × 10** ^**−5**^	2.03	0.99–4.16	0.0539
	rs78555439	A	C	1.24	0.80–1.90	0.3318	1.36	1.06–1.73	**0.0137**	1.45	0.62–3.43	0.3929
	rs2276767	A	C	1.17	0.74–1.87	0.4977	1.27	1.00–1.60	**0.0468**	1.32	0.52–3.32	0.5602

A1 = minor allele, A2 = major allele, CI = confidence interval, MetS = metabolic syndrome, OR = odds ratio, TGF-β = transforming growth factor-β. Analysis was performed with adjustment for covariates including age and sex. P values of < 0.05 are shown in bold.

Furthermore, as shown in Table 2, the association of two key SNPs, namely *SMAD2* rs11082639 and *TGFBR2* rs3773651, with MetS remained significant after applying Bonferroni correction (P < 0.05/(141 × 3) = 0.0001). As demonstrated in Table 2, for the *SMAD2* rs11082639 SNP, an increased risk of MetS was observed among subjects with MetS and those without MetS after adjustment for covariates such as age and sex for genetic models, including the additive model (odds ratio [OR] = 1.66; 95% confidence interval [CI] = 1.32–2.08; P = 1.4 × 10^−5^) and recessive model (OR = 2.82; 95% CI = 1.80–4.43; P = 6.6 × 10^−6^). Similarly, for the *TGFBR2* rs3773651 SNP, an increased risk of MetS was observed among the subjects after adjustment for covariates for genetic models, including the dominant model (OR = 1.59; 95% CI = 1.28–1.98; P = 3.1 × 10^−5^). In addition, the rs11082639, rs1981, rs10853560, and rs948603 SNPs of the *SMAD2* gene were found to be in strong LD (r^2^ > 0.8) to each other (Supplementary Table S2).

Table 3 shows the OR analysis of the association of two key SNPs, namely *SMAD2* rs11082639 and *TGFBR2* rs3773651, with the individual components of MetS: (a) high waist circumference vs. normal waist circumference; (b) high triglyceride vs. normal triglyceride; (c) low high-density lipoprotein (HDL) vs. normal HDL; (d) high blood pressure vs. normal blood pressure; and (e) high fasting glucose vs. normal fasting glucose. As shown in Table 3, for the *SMAD2* rs11082639 SNP, an increased risk of high waist circumference was observed among the subjects after adjustment for covariates for genetic models, including the additive model (OR = 1.47; 95% CI = 1.17–1.84; P = 0.0008) and recessive model (OR = 2.19; 95% CI = 1.40–3.43; P = 0.0006). The effect of *SMAD2* rs11082639 on high waist circumference remained significant after Bonferroni correction (P < 0.05/30 = 0.0017). In addition, we examined the association of the two implicated SNPs with MetS traits considered as continuous variables, including waist circumference, triglyceride, HDL, systolic blood pressure, diastolic blood pressure, and fasting glucose. As shown in Supplementary Table S4, the results suggest an association between *SMAD2* rs11082639 and MetS traits such as waist circumference (P = 0.0048) or fasting glucose (P = 0.0027).

**Table 3:** Covariate-adjusted odds ratio of the relationship between individual components of MetS and *SMAD2* rs11082639 and *TGFBR2* rs3773651 SNPs.

Individual components of the MetS	Additive model			Dominant model			Recessive model		
	OR	95% CI	P	OR	95% CI	P	OR	95% CI	P
(1) *SMAD2* rs11082639									
High waist circumference^a^	1.47	1.17–1.84	**0.0008**	1.03	0.88–1.21	0.6835	2.19	1.40–3.43	**0.0006**
High triglyceride^b^	1.25	0.98–1.59	0.0751	1.20	1.00–1.45	0.0530	1.47	0.91–2.38	0.1128
Low HDL^c^	1.29	1.02–1.61	0.0303	1.05	0.88–1.26	0.5848	1.65	1.05–2.59	0.0289
High blood pressure^d^	1.04	0.81–1.35	0.7429	1.00	0.83–1.21	0.9852	1.09	0.66–1.82	0.7347
High fasting glucose^e^	1.23	0.97–1.56	0.0846	0.98	0.81–1.18	0.8041	1.56	0.97–2.49	0.0668
(2) *TGFBR2* rs3773651									
High waist circumference^a^	1.11	0.80–1.53	0.5344	1.12	0.93–1.34	0.2252	1.20	0.63–2.29	0.5776
High triglyceride^b^	0.97	0.64–1.47	0.8856	1.16	0.93–1.44	0.1779	0.91	0.40–2.09	0.8230
Low HDL^c^	1.05	0.73–1.51	0.8038	1.27	1.04–1.56	0.0176	1.04	0.50–2.14	0.9185
High blood pressure^d^	1.13	0.76–1.68	0.5336	1.17	0.94–1.45	0.1649	1.25	0.57–2.73	0.5837
High fasting glucose^e^	1.07	0.72–1.58	0.7342	1.06	0.86–1.32	0.5691	1.13	0.52–2.46	0.7560

CI = confidence interval, HDL cholesterol = high-density lipoprotein cholesterol, MetS = metabolic syndrome, OR = odds ratio. Analysis was performed with adjustment for covariates including age and sex. P values of < 0.0017 (Bonferroni correction: 0.05/30) are shown in bold. ^a^Waist circumference ≥90 cm in male subjects, or waist circumference ≥80 cm in female subjects. ^b^Triglyceride ≥1.7 mmol/L. ^c^HDL <1.03 mmol/L in male subjects, or HDL <1.29 mmol/L in female subjects. ^d^Systolic blood pressure ≥130 mmHg or diastolic blood pressure ≥85 mmHg. ^e^Fasting glucose ≥5.6 mmol/L.

In addition, the generalized multifactor dimensionality reduction (GMDR) analysis was used to assess the effects of interaction between two key SNPs, namely *SMAD2* rs11082639 and *TGFBR2* rs3773651, on MetS and its individual components, with adjustment for the covariates of age and sex. Table 4 summarizes the results of GMDR analysis for two-way gene–gene interaction models with covariate adjustment to assess the effects of the two SNPs on MetS. As shown in Table 4, the two-way model involving *SMAD2* rs11082639 and *TGFBR2* rs3773651 was significant (P < 0.001). The effects of these two-way models remained significant after Bonferroni correction (P < 0.05/6 = 0.008). This finding indicated that a potential interaction between *SMAD2* and *TGFBR2* influences MetS. However, no two-way gene–gene interaction model influencing the individual components of MetS was obtained in this study.

**Table 4:** Two-way gene–gene interaction models by using the GMDR method with adjustment for age and sex.

Phenotype	Two-way interaction model	Testing accuracy (%)	P value
MetS	*SMAD2* rs11082639, *TGFBR2* rs3773651	54.55	**<0.001**
High waist circumference^a^	*SMAD2* rs11082639, *TGFBR2* rs3773651	50.68	0.278
High triglyceride^b^	*SMAD2* rs11082639, *TGFBR2* rs3773651	51.65	0.132
Low HDL^c^	*SMAD2* rs11082639, *TGFBR2* rs3773651	51.82	0.092
High blood pressure^d^	*SMAD2* rs11082639, *TGFBR2* rs3773651	49.77	0.606
High fasting glucose^e^	*SMAD2* rs11082639, *TGFBR2* rs3773651	49.46	0.664

GMDR = generalized multifactor dimensionality reduction, HDL cholesterol = high-density lipoprotein cholesterol, MetS = metabolic syndrome. P value was based on 1,000 permutations. Analysis was performed with adjustment for covariates including age and sex. P values of < 0.008 (Bonferroni correction: 0.05/6) are shown in bold. ^a^Waist circumference ≥90 cm in male subjects, or waist circumference ≥80 cm in female subjects. ^b^Triglyceride ≥1.7 mmol/L. ^c^HDL <1.03 mmol/L in male subjects, or HDL < 1.29 mmol/L in female subjects. ^d^Systolic blood pressure ≥130 mmHg or diastolic blood pressure ≥85 mmHg. ^e^Fasting glucose ≥5.6 mmol/L.

Furthermore, we utilized multivariable logistic regression analysis with adjustment for age and sex to assess the two-way *SMAD2* rs11082639 and *TGFBR2* rs3773651 interaction models selected by the GMDR method (Table 5). Our analysis revealed that subjects with the CC genotype of *SMAD2* rs11082639 and the AA genotype of *TGFBR2* rs3773651 had a 2.5-fold higher risk of MetS than those with the T allele of *SMAD2* rs11082639 and the AA genotype of *TGFBR2* rs3773651 (Table 5). Subjects with the CC genotype of *SMAD2* rs11082639 and the G allele of *TGFBR2* rs3773651 also had a 5.85-fold higher risk of MetS than those with the T allele of *SMAD2* rs11082639 and the AA genotype of *TGFBR2* rs3773651 (Table 5). Finally, subjects with the T allele of *SMAD2* rs11082639 and the G allele of *TGFBR2* rs3773651 had a 1.55-fold higher risk of MetS than those with the T allele of *SMAD2* rs11082639 and the AA genotype of *TGFBR2* rs3773651 (Table 5).

**Table 5:** Multivariable logistic regression analysis for the *SMAD2* rs11082639 and *TGFBR2* rs3773651 interaction model.

Two-way interaction model	OR	95% CI	P value^b^
*SMAD2* rs11082639 (TT + CT genotype) with*TGFBR2* rs3773651 (AA genotype)^a^	1		
*SMAD2* rs11082639 (CC genotype) with*TGFBR2* rs3773651 (AA genotype)	2.50	1.46–4.27	**0.0008**
*SMAD2* rs11082639 (CC genotype) with*TGFBR2* rs3773651 (GG + GA genotype)	5.85	2.48–13.78	**5.3 × 10** ^**−5**^
*SMAD2* rs11082639 (TT + CT genotype) with*TGFBR2* rs3773651 (GG + GA genotype)	1.55	1.23–1.94	**0.0002**

CI = confidence interval, OR = odds ratio. ^a^Reference. ^b^Versus reference. Analysis was performed with adjustment for covariates including age and sex. P values of < 0.05 are shown in bold.

Finally, statistical power analysis revealed that the present study had 99.9% power to detect associations of *SMAD2* rs11082639 and *TGFBR2* rs3773651 with MetS among the subjects with MetS and those without MetS.

## Discussion

To date, our association study is the first to examine whether 141 tag SNPs in 8 TGF-β signaling pathway-associated genes, namely *SMAD2*, *SMAD3*, *SMAD4*, *TGFB1*, *TGFB2*, *TGFB3*, *TGFBR1*, and *TGFBR2* genes, are significantly associated with the risk of MetS and its individual components independently, through gene–gene interactions, or both among Taiwanese individuals. Here, we report for the first time that the *SMAD2* and *TGFBR2* genes may play a key role in the development of MetS in a Taiwanese population. Notably, the significant association of two key SNPs, namely *SMAD2* rs11082639 and *TGFBR2* rs3773651, with MetS remained significant after Bonferroni correction (P < 0.0001). In addition, our data revealed that interactions between the *SMAD2* and *TGFBR2* genes may contribute to the etiology of MetS. Finally, our data revealed that the *SMAD2* rs11082639 SNP was associated with the individual component of MetS, namely high waist circumference.

In the present study, we found a positive association of MetS with five SNPs in the *SMAD2* gene, particularly the rs11082639 SNP. We also detected an association of *SMAD2* rs11082639 with the dichotomous categorical variable of high waist circumference and the continuous variables of waist circumference and fasting glucose. The *SMAD2* gene encodes the SMAD2 protein, which is recruited and phosphorylated by TGF-β receptors in response to TGF-β signals^1,2^. SMAD2 forms a heteromeric SMAD complex with SMAD4, which is subsequently translocated to the cell nucleus. Yang *et al*. reported that in *Smad2*-silenced cells in mice, gene expression related to lipogenesis was suppressed and gene expression related to β-oxidation was increased when Smad2 was inactivated by using an animal model of nonalcoholic steatohepatitis, one of the hepatic manifestations of MetS^12^. Their results indicated that the Smad signaling pathway is crucial for modulating lipid metabolism and lipid accumulation in hepatocytes through the suppression of lipogenesis-related genes and the induction of β-oxidation-related genes that promote the development of nonalcoholic steatohepatitis^12^. Several GWAS studies have been performed to investigate the genetic basis of MetS. For example, Kraja *et al*. performed a GWAS study on data from seven Caucasian cohorts and detected a significant association of triglyceride and glucose traits with the rs11820589 SNP in the BUD13 homolog (*BUD13*) gene and the rs12286037 SNP in the ZPR1 zinc finger (*ZPR1*) gene^23^. Moreover, another GWAS study by Kristiansson *et al*. implicated that the *ZPR1* rs964184 SNP may be associated with lipid traits such as triglyceride and HDL in Finnish cohorts^24^.

In the present study, we found an association of MetS with four SNPs in the *TGFBR2* gene, particularly the rs3773651 SNP. The *TGFBR2* gene encodes the TGF-β type II receptor, which is a member of the Ser/Thr protein kinase family and is a key mediator of TGF-β signaling transduction^1,2^. Several association studies have identified that the *TGFBR2* gene is associated with CAD^17,20^, congenital heart defects^18,19^, and various cancers^25^, which are some of the hallmarks of MetS. By analyzing glucose and insulin tolerance test results in an animal model of nonalcoholic steatohepatitis, Yang *et al*. demonstrated that silencing *Tgfbr2* may contribute to MetS manifestations, including weight gain and insulin resistance; their finding indicated that *Tgfbr2* is a potent mediator in the development of hepatic steatosis, hepatocyte death, inflammation, and fibrosis^12^.

Furthermore, we inferred the epistatic effects between the *SMAD2* and *TGFBR2* genes on MetS by using the GMDR approach. To the best of our knowledge, no other study has evaluated the interactions between these genes. In addition to determining the statistical significance of the interaction, we examined the potential biological mechanism underlying the interaction. The functional relevance of the interactive effects of *SMAD2* and *TGFBR2* on MetS remains to be elucidated. The *SMAD2* and *TGFBR2* genes encode the SMAD2 protein and TGF-β type II receptor, respectively, which are two core components of the TGF-β signaling pathway. Understanding the nature and extent of cross-talk between these two core components of the TGF-β signaling pathway is a critical future research direction. Our current understanding of the TGF-β signaling pathway is as follows^1,2^: First, the TGF-β ligand binds to the TGF-β type II receptor at the plasma membrane, resulting in the formation of the complex of the TGF-β type I receptor with the TGF-β type II receptor. Subsequently, the TGF-β type II receptor phosphorylates the TGF-β type I receptor. In turn, the activated TGF-β type I receptor phosphorylates SMAD2 and SMAD3 proteins. Finally, the phosphorylated SMAD2 and SMAD3 proteins form a complex with the SMAD4 protein, which is subsequently translocated into the nucleus for regulating the expression of specific target genes. Thus, regarding potential explanations for the biological effects of synergy between *SMAD2* and *TGFBR2*, we speculate that the *SMAD2* and *TGFBR2* gene products may participate in a common pathogenic pathway, that is the TGF-β signaling pathway, leading to MetS.

This study has some limitations. The main weakness is that our findings require much more research to verify if the observations are replicated in diversified ethnic populations^26–28^. Moreover, in this study, a subject with MetS could be mistakenly classified as a subject without MetS because no records on the medications prescribed for treatment of dyslipidaemia, hypertension or diabetes were available. In the Taiwan Biobank, individuals were asked in a questionnaire whether a doctor had ever told them they had certain diseases such as hyperlipidemia, hypertension or diabetes. Thus, we further removed control subjects with a self-reported diagnosis of hyperlipidemia, hypertension or diabetes and then investigated the association between MetS and two key SNPs, namely *SMAD2* rs11082639 and *TGFBR2* rs3773651. The association of these two implicated SNPs with MetS remained significant after applying Bonferroni correction (Supplementary Table S5). Based on the candidate gene approach, the current findings are considered as only preliminary owing to the absence of supporting evidence from larger hypothesis-free GWAS studies^23,24^. Additional prospective clinical trials including other ethnic groups and conducting GWAS studies are warranted to thoroughly evaluate the association and interactions of the investigated genes with MetS and its individual components^29–31^.

In conclusion, we conducted an extensive analysis of the association and interactions of the TGF-β signaling pathway-associated genes with MetS and its individual components in Taiwanese subjects. Our findings indicated that the *SMAD2* and *TGFBR2* genes may affect the prevalence of MetS independently and through complex gene–gene interactions. Furthermore, the TGF-β signaling pathway-associated genes may be associated with the components of MetS. These findings contribute to accumulating evidence supporting that the TGF-β signaling pathway influences MetS. Further investigation with larger sample sizes is essential to provide more insights into the role of the TGF-β signaling pathway-associated genes investigated in this study.

## Materials and Methods

### Study population

This study included Taiwanese subjects from the Taiwan Biobank. This biobank collected specimens and associated data from the general Taiwanese population with no history of cancer through recruitment centers across Taiwan during 2013–2015^32–39^. Recruitment centers encompass regional and municipal hospitals, where advertisements were posted to recruit potential participants and incentives, such as free general health examinations and travel fee reimbursement, were offered to the participants^32^. This biobank is mainly funded by the Taiwanese government and aims to provide researchers with opportunities for collaboration to facilitate public health-related research concerning local common chronic diseases^32^. The study cohort consisted of 3,000 subjects. Individuals who could perform activities of daily living, were aged 30–70 years, and were self-reported as being of Taiwanese Han Chinese ancestry were included in this study^33^. Individuals with a history of cancer or nonresidents of Taiwan were excluded^33^. Ethical approval for this study was granted by the Institutional Review Board of the Taiwan Biobank before conducting the study. Each subject signed an approved informed consent form. All experiments were performed in accordance with relevant guidelines and regulations.

### Metabolic syndrome

Measurements of metabolic traits including waist circumference, triglyceride, HDL cholesterol, systolic and diastolic blood pressure, and fasting glucose were obtained when subjects underwent general health examinations^32–34^. MetS was defined according to the International Diabetes Federation definition. Individual were considered to have MetS if they had central obesity (defined as waist circumference of ≥90 cm in male subjects and ≥80 cm in female subjects) and two or more of the following four components: (1) triglycerides ≥1.7 mmol/L; (2) HDL cholesterol <1.03 mmol/L in male subjects and <1.29 mmol/L in female subjects; (3) systolic blood pressure ≥130 mmHg or diastolic blood pressure ≥85 mmHg; and (4) fasting plasma glucose ≥5.6 mmol/L^40^. Two measurements of blood pressure were taken in both arms at least 10–15 minutes apart in the sitting position. These measurements were averaged to obtain the final blood pressure used in this study.

### Genotyping

DNA was isolated from blood samples using a QIAamp DNA blood kit following the manufacturer’s instructions (Qiagen, Valencia, CA, USA). The quality of the isolated genomic DNA was evaluated using agarose gel electrophoresis, and the quantity was determined using spectrophotometry^41^. SNP genotyping was performed using custom Taiwan Biobank chips and was achieved using the Axiom Genome-Wide Array Plate System (Affymetrix, Santa Clara, CA, USA). To efficiently obtain maximal genetic information from the samples of subjects with Taiwanese Han Chinese ancestry, the custom Taiwan Biobank chips were designed using SNPs with minor allele frequencies (MAFs) ≥5% on the Axiom Genome-Wide CHB 1 Array (Affymetrix, Inc.), using SNPs in exons with MAFs >10% on the Human Exome BeadChip (Illumina, Inc., San Diego, CA, USA), and using SNPs previously reported in ancestry information panels, cancer studies, and pharmacogenetic studies^33^.

In this study, the SNP panel covered 261 SNPs from the following eight TGF-β signaling pathway-associated genes: *SMAD2*, *SMAD3*, *SMAD4*, *TGFB1*, *TGFB2*, *TGFB3*, *TGFBR1*, and *TGFBR2* (Supplementary Table S1). Fifteen SNPs were excluded from further analysis due to failure to achieve the Hardy–Weinberg equilibrium (P < 0.05) or due to a genotyping call rate of <0.95. The genotyping results, including MAFs, P values for the Hardy–Weinberg equilibrium, and genotyping call rates, are shown in Supplementary Table S1. In addition, tag SNPs were identified using PLINK^42^, and an LD value (r²) of 0.8 was used as a threshold.

### Statistical analysis

Categorical data were evaluated using the χ^2^ test. The Student’s *t* test was used to compare the difference in the means calculated from two continuous variables. To evaluate the association of the investigated SNPs with MetS, we conducted a logistic regression analysis to estimate the ORs and their 95% CIs, adjusting for covariates including age and sex^43^. Furthermore, we evaluated the association of the investigated SNPs with individual components of MetS by using logistic regression analysis, adjusting for age and sex^44^. We assessed whether the genotype frequencies were in the Hardy–Weinberg equilibrium by using the χ^2^ goodness-of-fit test with 1 degree of freedom (i.e., the number of genotypes subtracted from the number of alleles). Multiple testing was adjusted using Bonferroni correction. The criterion for significance was set at P < 0.05 for all tests. Data are presented as mean ± standard deviation.

We employed the GMDR method to investigate gene–gene interactions^45^. We tested two-way interactions using 10-fold cross-validation. GMDR software provides some output parameters, including the testing accuracy and empirical P values, to assess each selected interaction. Moreover, we used age and sex as covariates for gene–gene interaction models in our interaction analyses. Permutation testing provides empirical P values of prediction accuracy as a benchmark based on 1,000 shuffles. To correct for multiple testing, we applied a conservative Bonferroni correction factor for the number of tests employed in the GMDR analysis.

Based on the effect sizes in this study, the power to detect significant associations was evaluated using QUANTO software (http://biostats.usc.edu/Quanto.html).

### Data Availability

The data that support the findings of this study are available from the Taiwan Biobank but restrictions apply to the availability of these data, which were used under license for the current study, and so are not publicly available. To apply for access to these third party data please contact the Taiwan Biobank.

## Electronic supplementary material

Supplementary Tables
