# Supplementary material for: Transforming growth factor-β signaling pathway-associated genes SMAD2 and TGFBR2 are implicated in metabolic syndrome in a Taiwanese population
Source: Sci Rep. 2017 Oct 19;7:13589. doi: 10.1038/s41598-017-14025-4 (PMC5648797; doi:10.1038/s41598-017-14025-4)
Supplement: Supplementary file 1 — Supplementary Tables [file 41598_2017_14025_MOESM1_ESM.pdf]

Supplementary Information for

Transforming growth factor-β signaling pathway-associated genes *SMAD2* and *TGFBR2* are implicated in metabolic syndrome in a Taiwanese population

Eugene Lin[1,2,3*], Po-Hsiu Kuo[4], Yu-Li Liu[5], Albert C. Yang[6,7], Shih-Jen Tsai[6,7*]

[1]Graduate Institute of Biomedical Sciences, China Medical University, Taichung, Taiwan

[2]Vita Genomics, Inc., Taipei, Taiwan

[3]TickleFish Systems Corporation, Seattle, WA, USA

[4]Department of Public Health, Institute of Epidemiology and Preventive Medicine, National Taiwan University, Taipei, Taiwan

[5]Center for Neuropsychiatric Research, National Health Research Institutes, Miaoli County, Taiwan

[6]Department of Psychiatry, Taipei Veterans General Hospital, Taipei, Taiwan

[7]Division of Psychiatry, National Yang-Ming University, Taipei, Taiwan

* *Corresponding author*:

E-mail: eugene.lin@vitagenomics.com (E.L.)

or

E-mail: tsai610913@gmail.com (S.-J.T.)

Short Title: Transforming growth factor-β and metabolic syndrome

Supplementary Table S1. Genotyping results for 261 SNPs in the TGF-β signaling pathway-associated genes of *SMAD2*, *SMAD3*, *SMAD4*, *TGFB1*, *TGFB2*, *TGFB3*, *TGFBR1*, and *TGFBR2*.

Gene	CHR	SNP	A1	A2	MAF	P (HWE)	Genotyping call rate
SMAD2	18	rs1981	A	G	0.185	0.078	0.997
		rs1792666	A	T	0.369	0.255	0.999
		rs79502327	T	C	0.173	0.201	0.995
		rs1792684	A	G	0.372	0.309	0.999
		rs62086544	C	A	0.183	**0.037**	0.995
		rs1792658	C	A	0.457	0.358	0.999
		rs1792675	G	T	0.187	**0.047**	0.998
		rs74430094	C	T	0.055	0.112	0.999
		rs10853560	C	T	0.183	0.143	0.997
		rs16958602	C	T	0.137	0.588	0.998
		rs11082639	C	T	0.184	0.222	0.994
		rs12458238	T	G	0.365	0.608	0.996
		rs948603	G	A	0.197	0.225	0.999
		rs118083713	G	A	0.055	0.284	0.999
		rs4940086	C	T	0.419	0.154	0.999
		rs142886105	T	G	0.085	0.906	0.998
		rs12457664	G	A	0.343	0.418	0.999
		rs4939672	C	G	0.169	0.362	1.000
		rs4940102	A	C	0.340	0.393	0.999
		rs2000709	A	G	0.169	0.559	1.000
SMAD3	15	rs12904944	A	G	0.422	0.216	0.998
		rs11632964	T	C	0.316	0.899	1.000
		rs12901071	G	A	0.207	0.617	0.999
		rs12901499	A	G	0.482	0.256	0.997
		rs16950556	G	C	0.155	0.529	0.999
		rs72743423	C	T	0.263	0.109	0.999
		rs12910698	T	G	0.364	0.134	0.999
		rs72743427	G	A	0.254	0.193	1.000

		rs12907997	T	C	0.380	0.174	0.996
		rs4776339	C	T	0.386	0.114	0.999
		rs74485833	T	C	0.216	0.146	1.000
		rs79826987	A	G	0.075	0.236	0.999
		rs7176870	G	A	0.203	0.101	0.999
		rs28417316	G	A	0.075	0.146	0.999
		rs7181878	A	G	0.370	0.108	0.999
		rs75029750	C	A	0.173	**0.000**	0.958
		rs9972423	A	T	0.385	0.877	0.998
		rs4776890	G	T	0.233	0.239	0.999
		rs77035000	A	G	0.150	0.615	0.999
		rs17213990	A	G	0.075	0.146	0.998
		rs56336169	A	G	0.075	0.067	0.997
		rs79887171	A	G	0.107	0.774	1.000
		rs118091321	A	G	0.150	0.615	0.999
		rs2118611	T	C	0.467	0.942	0.998
		rs62006018	T	C	0.243	0.519	0.998
		rs7359174	A	G	0.470	0.420	0.999
		rs17214419	T	A	0.208	0.470	0.999
		rs16950635	A	G	0.278	0.219	0.999
		rs71398296	T	C	0.278	0.220	0.999
		rs6494633	T	C	0.078	0.126	0.995
		rs6494634	T	C	0.287	1.000	0.997
		rs12102171	T	C	0.352	0.138	0.998
		rs67614233	T	C	0.206	0.577	0.999
		rs4147358	C	A	0.371	0.876	1.000
		rs6494635	A	G	0.081	1.000	0.998
		rs2414937	C	G	0.360	**0.004**	0.999
		rs12915039	C	A	0.300	0.409	0.997
		rs745103	A	G	0.403	0.820	0.998
		rs17293443	C	T	0.055	0.477	0.999
		rs72743459	T	C	0.105	0.173	0.999
		rs2289263	G	T	0.354	0.631	0.998
		rs2033785	G	C	0.393	0.939	0.997
		rs11637659	A	G	0.300	0.828	0.999
		rs17293632	T	C	0.025	0.428	0.999

		rs744910	A	G	0.393	0.909	0.998
		rs2033784	G	A	0.307	0.699	0.998
		rs4776900	T	C	0.264	0.707	0.999
		rs4601989	T	C	0.390	1.000	0.997
		rs17228212	C	T	0.028	1.000	0.961
		rs11639295	T	C	0.429	0.882	1.000
		rs12913547	C	T	0.386	0.355	0.998
		rs12708492	C	T	0.390	0.055	0.998
		rs12324906	A	G	0.271	0.746	1.000
		rs3784679	A	G	0.298	0.662	0.998
		rs1470002	G	A	0.247	0.492	0.999
		rs12909923	A	G	0.083	**0.000**	0.964
		rs12595334	T	C	0.478	0.884	0.999
		rs11556090	G	A	0.209	**0.020**	0.999
		rs3743343	C	T	0.279	0.717	0.999
		rs1052488	C	T	0.278	1.000	0.996
SMAD4	18	rs12958604	G	A	0.483	0.214	0.998
		rs17811426	T	C	0.420	0.881	0.998
		rs12456284	G	A	0.370	0.638	0.999
		rs2282544	C	G	0.067	0.659	0.998
TGFB1	19	rs2241715	C	A	0.436	0.127	0.997
TGFB2	1	rs10482724	A	G	0.149	0.773	0.998
		rs2799098	G	A	0.196	0.201	0.998
		rs17047682	G	A	0.131	0.809	0.994
		rs1417488	T	C	0.369	0.455	0.995
		rs11581605	G	A	0.281	0.618	0.995
		rs17047703	A	C	0.278	0.617	0.999
		rs6662137	G	T	0.371	0.291	0.999
		rs12094199	G	A	0.092	0.383	1.000
		rs3892225	G	A	0.172	0.700	0.997
		rs2009112	T	C	0.147	0.611	0.998
		rs10482750	C	T	0.187	0.505	0.987
		rs2799086	T	C	0.286	0.264	1.000
		rs10482751	C	T	0.495	0.381	0.999
		rs17047740	T	C	0.110	0.577	0.999
		rs2027566	C	A	0.499	0.093	0.999

		rs2027567	G	A	0.388	0.538	0.997
		rs10863396	G	A	0.499	0.073	0.998
		rs12058490	G	A	0.113	0.522	0.999
		rs2796814	G	C	0.377	0.305	0.970
		rs12405215	T	C	0.164	0.142	0.999
		rs947712	C	T	0.489	0.942	0.996
		rs947711	G	C	0.335	0.251	0.999
		rs1539399	A	G	0.416	0.735	0.998
		rs76916003	A	G	0.141	0.328	0.998
		rs2799090	G	A	0.336	0.130	0.999
		rs79839744	T	C	0.169	0.363	0.997
		rs6703224	T	C	0.170	0.196	0.999
		rs1317681	A	G	0.493	0.635	0.998
		rs1891467	A	G	0.327	0.245	1.000
		rs2796819	A	G	0.264	0.814	0.997
		rs1797071	T	C	0.438	0.578	0.999
		rs2799083	C	T	0.214	0.415	0.998
		rs17047804	T	C	0.480	0.826	0.998
		rs10863397	T	C	0.172	0.223	0.999
		rs2796821	T	C	0.213	0.624	0.998
		rs2796822	A	G	0.265	0.963	0.999
		rs2796823	T	A	0.213	0.586	0.998
		rs2000220	A	G	0.266	0.925	0.997
		rs2796813	T	C	0.265	0.851	0.999
		rs6657275	A	G	0.279	0.618	0.999
		rs1342586	C	T	0.455	0.416	0.989
		rs4846479	G	T	0.281	0.717	0.998
		rs77246858	C	T	0.084	0.721	0.999
		rs6658473	C	T	0.282	0.464	0.968
		rs10482792	G	A	0.278	0.785	0.998
		rs10482796	T	C	0.452	0.580	0.999
		rs1418553	C	T	0.277	0.649	0.999
		rs900	A	T	0.272	0.373	0.967
		rs991967	A	C	0.277	0.681	0.998
TGFB3	14	rs3917211	C	T	0.353	0.162	1.000
		rs3917210	T	A	0.356	0.247	0.996

		rs2284791	C	G	0.423	0.501	0.999
		rs3917205	A	G	0.112	0.519	1.000
		rs2359994	C	A	0.449	0.160	0.997
		rs3917201	C	T	0.495	0.214	0.998
		rs3917192	T	C	0.438	0.629	0.996
		rs4252328	T	C	0.441	0.459	0.998
		rs2268624	G	C	0.396	0.939	0.999
		rs2268625	C	T	0.397	1.000	0.998
		rs2284792	G	A	0.420	0.911	0.999
		rs3917158	T	C	0.368	0.906	0.996
		rs2268626	C	T	0.331	0.283	0.998
		rs3917148	G	T	0.100	0.068	0.998
TGFBR1	9	rs7874221	C	T	0.437	0.373	0.998
		rs6478972	A	G	0.475	**0.008**	0.994
		rs10760670	A	G	0.467	0.142	0.999
		rs10760672	T	C	0.434	0.352	0.997
		rs10819638	T	C	0.435	0.353	0.999
		rs6478974	A	T	0.325	0.480	0.999
		rs10739778	C	A	0.470	0.175	0.998
		rs10988713	T	C	0.468	0.123	0.996
		rs10512263	C	T	0.231	0.279	0.998
		rs11568764	C	T	0.063	0.215	0.996
		rs10988719	A	T	0.228	0.378	0.998
		rs7042852	A	T	0.445	**0.001**	0.984
		rs334353	G	T	0.437	0.414	0.998
		rs334354	A	G	0.436	0.373	1.000
		rs334355	A	G	0.469	0.602	0.969
		rs334348	G	A	0.473	0.175	0.999
		rs334349	A	G	0.479	0.634	0.998
		rs1590	G	T	0.472	0.199	0.997
TGFBR2	3	rs764522	G	C	0.089	**0.043**	0.999
		rs6550004	C	A	0.101	0.841	0.998
		rs6550005	A	G	0.100	0.478	0.999
		rs6770038	T	C	0.092	0.583	1.000
		rs1835538	A	G	0.125	0.315	0.999
		rs56402737	T	C	0.107	0.775	0.999

		rs2163417	T	C	0.127	0.934	0.996
		rs1991657	C	T	0.233	0.330	0.993
		rs9310938	C	T	0.243	0.653	0.989
		rs9790268	T	C	0.415	0.098	0.999
		rs9790292	T	C	0.414	0.055	0.997
		rs1036095	C	G	0.234	0.839	0.999
		rs9881945	T	G	0.124	**0.043**	0.999
		rs6773330	G	A	0.359	1.000	0.999
		rs1864616	A	G	0.191	0.140	1.000
		rs11709624	C	G	0.308	0.932	0.997
		rs17025785	T	C	0.302	0.664	0.997
		rs4522809	G	A	0.290	0.506	0.999
		rs34767766	G	A	0.101	0.269	0.999
		rs5020833	G	C	0.447	0.796	0.996
		rs17025788	C	G	0.234	1.000	1.000
		rs6809777	T	C	0.190	0.373	1.000
		rs12490899	T	C	0.255	0.736	0.997
		rs17025824	G	A	0.252	0.923	0.999
		rs12491780	T	C	0.290	0.657	0.998
		rs1431131	A	T	0.306	0.195	0.990
		rs2043138	G	C	0.196	**0.009**	0.990
		rs6775211	G	C	0.190	0.075	0.999
		rs12487185	A	G	0.312	0.394	0.997
		rs76016701	G	A	0.165	0.791	0.999
		rs11924422	C	A	0.278	0.649	0.998
		rs13083813	A	T	0.269	0.777	0.964
		rs11129421	T	C	0.271	1.000	0.998
		rs12495646	C	A	0.304	1.000	0.999
		rs3863057	T	C	0.203	0.308	0.992
		rs12493607	G	C	0.299	0.861	0.997
		rs9823731	A	G	0.267	1.000	0.999
		rs1155705	A	G	0.302	0.385	0.992
		rs1155708	G	A	0.294	0.505	0.982
		rs74425972	C	T	0.097	**0.021**	0.998
		rs57037798	G	A	0.297	0.965	0.999
		rs58965035	C	T	0.300	0.759	0.981

		rs13086588	G	T	0.438	0.436	0.998
		rs2082224	A	G	0.409	0.473	0.999
		rs891595	G	A	0.388	0.442	0.999
		rs1078985	G	A	0.158	0.215	0.999
		rs11466500	T	C	0.386	0.203	0.997
		rs1036097	T	C	0.457	0.854	0.998
		rs1036096	A	G	0.381	0.461	0.997
		rs76042225	G	A	0.075	0.791	0.998
		rs3773634	G	A	0.156	0.367	0.999
		rs10510636	A	G	0.380	0.416	0.999
		rs3773636	T	C	0.388	0.395	0.991
		rs995435	A	G	0.319	0.256	0.999
		rs6792117	G	A	0.121	0.301	0.992
		rs4583693	G	A	0.173	0.160	0.999
		rs749794	A	G	0.291	0.929	0.999
		rs3773641	T	G	0.291	0.757	0.998
		rs3773642	T	C	0.282	0.684	0.995
		rs76248649	A	G	0.172	0.199	0.997
		rs3773643	G	A	0.176	**0.005**	0.963
		rs12498079	T	A	0.229	**0.000**	1.000
		rs3773644	T	C	0.429	0.218	0.998
		rs3773645	G	C	0.316	0.704	0.997
		rs11466511	C	A	0.154	0.399	0.996
		rs11466512	A	T	0.317	0.726	**0.923**
		rs2228048	T	C	0.316	0.704	0.998
		rs11466515	C	A	0.317	0.736	0.999
		rs11466521	T	C	0.155	0.292	0.984
		rs3773649	A	G	0.318	0.674	0.999
		rs75862500	G	T	0.100	0.761	1.000
		rs2116142	G	A	0.324	0.803	0.999
		rs3773650	A	C	0.299	0.177	1.000
		rs3773651	G	A	0.113	0.785	1.000
		rs3773652	A	G	0.480	0.660	0.995
		rs3821671	A	G	0.306	0.731	0.999
		rs78555439	A	C	0.091	0.076	0.999
		rs2372212	A	G	0.356	0.936	0.997

		rs1346907	A	G	0.317	0.354	0.999
		rs876687	C	T	0.319	0.737	0.999
		rs876688	A	G	0.250	0.143	0.998
		rs877572	G	C	0.329	0.836	0.997
		rs3773660	A	G	0.319	0.867	0.998
		rs3773661	C	G	0.320	0.834	0.999
		rs73054849	A	C	0.319	0.705	0.997
		rs1367609	T	G	0.432	0.793	0.985
		rs9843143	G	C	0.435	0.231	0.984
		rs11466522	A	G	0.316	0.833	0.998
		rs9843942	A	G	0.371	0.368	0.999
		rs2276768	T	C	0.400	0.119	0.999
		rs3773663	G	A	0.494	0.381	0.999
		rs304839	T	A	0.128	0.682	0.999
		rs12637406	G	A	0.367	0.157	0.999
		rs2276767	A	C	0.101	0.688	1.000
		rs744751	A	G	0.101	0.688	1.000

A1 = minor allele, A2 = major allele, Chr = chromosome, HWE = Hardy–Weinberg equilibrium, MAF = minor allele frequency, TGF-β = transforming growth factor-β.

P values of < 0.05 are shown in bold.

Genotyping call rate values of < 0.95 are shown in bold.

Supplementary Table S2. 141 tag SNPs in the TGF-β signaling pathway-associated genes of *SMAD2*, *SMAD3*, *SMAD4*, *TGFB1*, *TGFB2*, *TGFB3*, *TGFBR1*, and *TGFBR2*.

Gene	CHR	SNP	With other SNPs in strong LD ($r^2 > 0.8$)
SMAD2	18	rs79502327	
		rs1792684	rs1792666, rs12458238, rs12457664, rs4940102
		rs1792658	
		rs74430094	rs118083713
		rs16958602	
		rs11082639	rs1981, rs10853560, rs948603
		rs4940086	
		rs142886105	
		rs2000709	rs948603, rs4939672
SMAD3	15	rs12904944	
		rs11632964	
		rs12901071	
		rs12901499	
		rs16950556	
		rs72743423	rs72743427
		rs12907997	
		rs74485833	rs7176870
		rs28417316	rs79826987
		rs7181878	rs12910698, rs4776339
		rs9972423	
		rs4776890	
		rs77035000	rs118091321
		rs17213990	rs56336169
		rs79887171	
		rs2118611	
		rs62006018	
		rs7359174	
		rs16950635	rs71398296
		rs6494633	
		rs6494634	
		rs12102171	

		rs67614233	rs17214419
		rs4147358	
		rs6494635	
		rs12915039	
		rs745103	
		rs17293443	
		rs72743459	
		rs2289263	
		rs2033785	rs744910, rs4601989, rs11639295, rs12913547, rs12708492
		rs11637659	rs4776900
		rs17293632	
		rs2033784	
		rs17228212	
		rs12324906	
		rs3784679	
		rs1470002	
		rs12595334	
		rs3743343	rs1052488
SMAD4	18	rs12958604	
		rs17811426	rs12456284
		rs2282544	
TGFB1	19	rs2241715	
TGFB2	1	rs10482724	
		rs2799098	
		rs17047682	
		rs1417488	
		rs11581605	rs17047703
		rs6662137	
		rs12094199	
		rs3892225	
		rs2009112	
		rs10482750	
		rs2799086	
		rs10482751	rs2027566, rs10863396
		rs17047740	

		rs2027567	
		rs12058490	
		rs2796814	
		rs12405215	rs79839744, rs6703224, rs10863397
		rs947712	
		rs947711	rs2799090
		rs1539399	
		rs76916003	
		rs1317681	rs17047804
		rs1891467	
		rs2796819	rs2796822, rs2000220, rs2796813, rs6657275, rs1418553, rs991967
		rs1797071	rs1342586, rs10482796
		rs2799083	rs2796821, rs2796823
		rs4846479	rs6658473, rs10482792, rs900
		rs77246858	
TGFB3	14	rs3917211	rs3917210
		rs2284791	
		rs3917205	
		rs3917201	rs2359994, rs3917192
		rs4252328	rs2268624, rs2268625, rs2284792, rs3917158
		rs2268626	
		rs3917148	
TGFBR1	9	rs7874221	rs10760670, rs10760672, rs10819638, rs10739778, rs10988713, rs334353, rs334354, rs334355, rs334348, rs334349, rs1590
		rs6478974	
		rs10512263	rs10988719
		rs11568764	
TGFBR2	3	rs6550004	rs6550005, rs6770038
		rs1835538	
		rs56402737	rs2163417
		rs1991657	rs9310938
		rs9790268	rs9790292
		rs1036095	
		rs6773330	

		rs1864616	
		rs11709624	rs17025785, rs4522809
		rs34767766	
		rs5020833	
		rs17025788	
		rs6809777	
		rs12490899	rs17025824
		rs12491780	
		rs1431131	rs12487185, rs11924422, rs12495646, rs12493607, rs1155705, rs1155708, rs57037798, rs58965035, rs9823731
		rs6775211	
		rs76016701	
		rs13083813	rs11129421
		rs3863057	
		rs13086588	rs2082224
		rs891595	rs11466500, rs1036096, rs10510636, rs3773636
		rs1078985	rs3773634
		rs1036097	
		rs76042225	
		rs995435	
		rs6792117	
		rs4583693	rs76248649, rs11466511, rs11466521
		rs749794	rs3773641
		rs3773642	
		rs3773644	
		rs3773645	rs11466515, rs3773649, rs2116142
		rs2228048	
		rs75862500	
		rs3773650	
		rs3773651	
		rs3773652	
		rs3821671	
		rs78555439	

		rs2372212	
		rs1346907	rs877572
		rs876687	rs3773660, rs3773661, rs73054849, rs11466522
		rs876688	
		rs1367609	rs9843143
		rs9843942	
		rs2276768	rs12637406
		rs3773663	
		rs304839	
		rs2276767	rs744751

Chr = chromosome, LD = linkage disequilibrium, TGF-β = transforming growth factor-β.

Supplementary Table S3. Covariate-adjusted odds ratio analysis of the relationship between MetS and 141 tag SNPs in the TGF-β signaling pathway-associated genes of *SMAD2*, *SMAD3*, *SMAD4*, *TGFB1*, *TGFB2*, *TGFB3*, *TGFBR1*, and *TGFBR2*.

Gene	CHR	SNP	A1	A2	P (Additive)	P (Dominant)	P (Recessive)
SMAD2	18	rs79502327	T	C	0.9444	0.9944	0.9419
		rs1792684	A	G	**0.0352**	0.4477	**0.0229**
		rs1792658	C	A	0.2314	0.4537	0.2386
		rs74430094	C	T	**0.0480**	0.0562	0.0536
		rs16958602	C	T	0.6549	0.8428	0.6592
		rs11082639	C	T	**$1.4x10^{-5}$**	0.6611	**$6.6\ x10^{-6}$**
		rs4940086	C	T	**0.0015**	**0.0052**	**0.0102**
		rs142886105	T	G	0.9035	0.8422	0.9085
		rs2000709	A	G	**0.0020**	0.9567	**0.0013**
SMAD3	15	rs12904944	A	G	0.2549	0.4759	0.2790
		rs11632964	T	C	0.2696	0.2435	0.3921
		rs12901071	G	A	0.4246	0.5247	0.4614
		rs12901499	A	G	0.5321	0.9457	0.3536
		rs16950556	G	C	0.5127	0.3543	0.5636
		rs72743423	C	T	0.9300	0.1872	0.8068
		rs12907997	T	C	0.4078	0.1165	0.8086
		rs74485833	T	C	0.0751	0.5851	0.0707
		rs28417316	G	A	0.4207	**0.0099**	0.4637
		rs7181878	A	G	**0.0421**	0.0725	0.0989
		rs9972423	A	T	0.0782	0.1302	0.1442
		rs4776890	G	T	0.2498	0.1790	0.3239
		rs77035000	A	G	0.6936	0.0965	0.7931
		rs17213990	A	G	0.3490	0.7292	0.3440
		rs79887171	A	G	0.6653	0.3479	0.6949
		rs2118611	T	C	0.0703	0.2481	0.0825
		rs62006018	T	C	0.8742	0.2157	0.9248
		rs7359174	A	G	0.4682	0.7035	0.4333
		rs16950635	A	G	0.3830	0.4159	0.2197
		rs6494633	T	C	0.1121	0.8660	0.1095

		rs6494634	T	C	0.2230	0.9720	0.1628
		rs12102171	T	C	0.5188	0.9559	0.4461
		rs67614233	T	C	**0.0469**	0.2455	0.0568
		rs4147358	C	A	0.4803	0.9596	0.3872
		rs6494635	A	G	0.4864	0.0692	0.5156
		rs12915039	C	A	0.0599	0.5121	0.0507
		rs745103	A	G	0.3591	0.4254	0.4625
		rs17293443	C	T	0.9978	0.4018	0.9978
		rs72743459	T	C	0.1694	0.6844	0.1699
		rs2289263	G	T	**0.0340**	0.9775	**0.0085**
		rs2033785	G	C	0.4088	0.9083	0.2622
		rs11637659	A	G	0.9652	0.7060	0.8653
		rs17293632	T	C	0.9988	0.3175	0.9988
		rs2033784	G	A	0.7854	0.5878	0.6182
		rs17228212	C	T	0.9991	0.0647	0.9991
		rs12324906	A	G	0.7649	0.1930	0.5020
		rs3784679	A	G	0.9211	0.6759	0.8051
		rs1470002	G	A	0.6323	0.4590	0.4989
		rs12595334	T	C	0.6786	0.5938	0.8722
		rs3743343	C	T	0.6698	0.9806	0.6302
SMAD4	18	rs12958604	G	A	0.2003	0.8351	0.0778
		rs17811426	T	C	0.0999	0.9625	**0.0315**
		rs2282544	C	G	0.9270	0.0583	0.9707
TGFB1	19	rs2241715	C	A	0.3200	0.7348	0.1051
TGFB2	1	rs10482724	A	G	0.9713	0.3732	0.9626
		rs2799098	G	A	0.4870	0.2547	0.5818
		rs17047682	G	A	0.1086	0.0769	0.1274
		rs1417488	T	C	0.7175	0.4000	0.9620
		rs11581605	G	A	1.0000	0.2901	0.7655
		rs6662137	G	T	0.2689	0.4511	0.3138
		rs12094199	G	A	0.1732	0.5214	0.1603
		rs3892225	G	A	0.5740	0.9598	0.5644
		rs2009112	T	C	0.3422	0.7512	0.3154
		rs10482750	C	T	0.9014	0.5371	0.8324
		rs2799086	T	C	0.5320	**0.0378**	0.9014
		rs10482751	C	T	0.2201	0.2074	0.4671

		rs17047740	T	C	0.9901	0.1280	0.9252
		rs2027567	G	A	0.9162	0.3438	0.7012
		rs12058490	G	A	0.6732	0.2793	0.7117
		rs2796814	G	C	0.8945	0.3936	0.7923
		rs12405215	T	C	0.7046	0.8432	0.7108
		rs947712	C	T	0.4871	0.6300	0.5153
		rs947711	G	C	0.4649	0.7523	0.4579
		rs1539399	A	G	0.8845	0.5832	0.8774
		rs76916003	A	G	0.2495	0.8701	0.2371
		rs1317681	A	G	0.8614	0.9838	0.7952
		rs1891467	A	G	0.6673	0.8632	0.6572
		rs2796819	A	G	0.6300	0.7582	0.6508
		rs1797071	T	C	0.8529	0.8242	0.6745
		rs2799083	C	T	0.3576	0.6498	0.2947
		rs4846479	G	T	0.6174	0.8247	0.5345
		rs77246858	C	T	0.3977	0.2791	0.4124
TGFB3	14	rs3917211	C	T	0.3021	**0.0197**	0.8500
		rs2284791	C	G	0.1661	**0.0018**	0.8127
		rs3917205	A	G	0.1144	0.0989	0.1356
		rs3917201	C	T	**0.0190**	**0.0012**	0.6010
		rs4252328	T	C	0.0553	**0.0151**	0.3682
		rs2268626	C	T	**0.0142**	0.0643	**0.0301**
		rs3917148	G	T	0.9641	0.4742	0.9974
TGFBR1	9	rs7874221	C	T	0.2276	0.2516	0.3832
		rs6478974	A	T	0.9812	0.4333	0.7463
		rs10512263	C	T	0.8425	0.8713	0.8561
		rs11568764	C	T	0.9516	0.5930	0.9632
TGFBR2	3	rs6550004	C	A	0.2971	0.5300	0.3042
		rs1835538	A	G	0.7152	0.1711	0.7780
		rs56402737	T	C	0.2640	0.1405	0.2881
		rs1991657	C	T	0.1309	**0.0241**	0.2320
		rs9790268	T	C	0.5681	0.8826	0.4973
		rs1036095	C	G	0.9325	0.2819	0.8957
		rs6773330	G	A	0.7469	0.5429	0.5048
		rs1864616	A	G	0.6397	0.0802	0.8232
		rs11709624	C	G	0.6600	0.5021	0.7823

		rs34767766	G	A	0.7684	0.1943	0.7127
		rs5020833	G	C	0.5664	0.8643	0.3338
		rs17025788	C	G	0.9737	0.7473	0.9216
		rs6809777	T	C	0.6636	0.4951	0.7157
		rs12490899	T	C	0.7898	0.6846	0.7045
		rs12491780	T	C	0.2711	0.6158	0.2756
		rs1431131	A	T	0.1858	0.6255	0.1751
		rs6775211	G	C	0.1731	0.6316	0.1759
		rs76016701	G	A	0.7206	0.9717	0.7087
		rs13083813	A	T	0.9615	0.2206	0.7877
		rs3863057	T	C	0.9345	0.3796	0.9613
		rs13086588	G	T	0.8734	0.6506	0.5778
		rs891595	G	A	0.4735	0.4567	0.1779
		rs1078985	G	A	0.6522	0.9237	0.6501
		rs1036097	T	C	0.7813	0.8995	0.7590
		rs76042225	G	A	0.2765	0.3822	0.2865
		rs995435	A	G	0.5462	0.8859	0.4501
		rs6792117	G	A	0.1115	0.8656	0.1061
		rs4583693	G	A	0.2575	0.1050	0.3297
		rs749794	A	G	0.3807	0.4137	0.4611
		rs3773642	T	C	0.9006	0.2655	0.8583
		rs3773644	T	C	0.0784	0.0750	0.2143
		rs3773645	G	C	0.9809	0.9328	0.9541
		rs2228048	T	C	0.3650	0.3900	0.4515
		rs75862500	G	T	0.0811	0.1250	0.0929
		rs3773650	A	C	0.7494	0.5052	0.8780
		rs3773651	G	A	**0.0285**	**3.1 x10^{-5}**	0.0539
		rs3773652	A	G	0.4486	0.7239	0.3925
		rs3821671	A	G	0.6557	0.5725	0.7435
		rs78555439	A	C	0.3318	**0.0137**	0.3929
		rs2372212	A	G	0.5213	0.1736	0.8526
		rs1346907	A	G	0.4920	0.4108	0.6125
		rs876687	C	T	0.6682	0.7807	0.5517
		rs876688	A	G	0.5518	0.7227	0.5689
		rs1367609	T	G	0.8430	0.3934	0.7438
		rs9843942	A	G	0.2165	0.4637	0.2396

		rs2276768	T	C	0.6466	0.4906	0.8393
		rs3773663	G	A	0.5725	0.9824	0.3832
		rs304839	T	A	0.4513	0.7442	0.4267
		rs2276767	A	C	0.4977	**0.0468**	0.5602

A1 = minor allele, A2 = major allele, Chr = chromosome, MetS = metabolic syndrome,

TGF-β = transforming growth factor-β.

P values of < 0.05 are shown in bold.

Analysis was performed with adjustment for covariates including age and sex.

Supplementary Table S4. Linear regression models of associations between individual components of MetS and *SMAD2* rs11082639 and *TGFBR2* rs3773651 SNPs.

Individual components of the MetS	Additive model			Dominant model			Recessive model		
	BETA	SE	P	BETA	SE	P	BETA	SE	P
(1) *SMAD2* rs11082639									
Waist circumference	1.33	0.49	**0.0065**	0.005	0.35	0.9893	2.75	0.97	**0.0048**
Triglyceride	2.93	4.63	0.5268	2.69	3.33	0.4184	5.11	9.20	0.5786
HDL	-0.53	0.67	0.4272	-0.43	0.48	0.3651	-0.94	1.32	0.4775
Diastolic blood pressure	0.59	0.54	0.2803	-0.21	0.39	0.5957	1.28	1.08	0.2349
Systolic blood pressure	1.45	0.81	0.0724	-0.05	0.58	0.9355	3.01	1.60	0.0607
Fasting glucose	3.32	1.14	**0.0035**	0.20	0.82	0.8088	6.78	2.26	**0.0027**
(2) *TGFBR2* rs3773651									
Waist circumference	0.69	0.73	0.3425	0.61	0.41	0.1358	1.27	1.45	0.3830
Triglyceride	8.43	6.84	0.2179	3.63	3.82	0.3419	16.29	13.65	0.2328
HDL	-0.16	0.99	0.8738	-0.37	0.55	0.4979	-0.24	1.97	0.9046
Diastolic blood pressure	0.88	0.80	0.2719	0.04	0.45	0.9213	1.78	1.60	0.2671
Systolic blood pressure	1.58	1.20	0.1855	1.31	0.67	0.0501	2.92	2.39	0.2209
Fasting glucose	2.60	1.68	0.1220	0.38	0.94	0.6860	5.19	3.36	0.1222

BETA = Beta coefficients, HDL cholesterol = high-density lipoprotein cholesterol, MetS = metabolic syndrome, SE = standard error.

Analysis was performed with adjustment for covariates including age and sex.

P values of < 0.05 are shown in bold.

Supplementary Table S5. Covariate-adjusted odds ratio analysis of the relationship between MetS and *SMAD2* rs11082639 and *TGFBR2* rs3773651 SNPs by removing control subjects with a self-reported diagnosis of hyperlipidemia, hypertension or diabetes.

				Additive model			Dominant model			Recessive model		
Gene	SNP	A1	A2	OR	95% CI	P	OR	95% CI	P	OR	95% CI	P
SMAD2	rs11082639	C	T	1.65	1.30-2.09	**4.1 x 10^{-5}**	1.07	0.87-1.32	0.4996	2.76	1.72-4.44	**2.5 x 10^{-5}**
TGFBR2	rs3773651	G	A	1.47	1.01-2.13	0.0430	1.52	1.21-1.91	**2.6 x 10^{-4}**	1.97	0.94-4.13	0.0732

A1 = minor allele, A2 = major allele, CI = confidence interval, MetS = metabolic syndrome, OR = odds ratio.

Analysis was performed with adjustment for covariates including age and sex. P values of < 0.008 (Bonferroni correction: 0.05/(2 × 3)) are shown in bold.

The number of control subjects with a self-reported diagnosis of hyperlipidemia, hypertension or diabetes was 87, 166 or 67, respectively.

The sample size was 2,714 after removing control subjects with a self-reported diagnosis of hyperlipidemia, hypertension or diabetes.
